# The effects of meteorological factors on influenza among children in Guangzhou, China

**DOI:** 10.1111/irv.12617

**Published:** 2018-12-13

**Authors:** Qiaozhi Guo, Zhiqiang Dong, Weilin Zeng, Wenjun Ma, Danyang Zhao, Xin Sun, Sitang Gong, Jianpeng Xiao, Tiegang Li, Wensui Hu

**Affiliations:** ^1^ Guangzhou Women and Children's Medical Center Guangzhou Medical University Guangzhou China; ^2^ Guangzhou Center for Disease Control and Prevention Guangzhou China; ^3^ Guangdong Provincial Institute of Public Health Guangdong Provincial Center for Disease Control and Prevention Guangzhou China

**Keywords:** Children, distributed lag nonlinear model, Influenza, meteorological factors

## Abstract

**Background:**

Influenza seriously affects the health of children, yet little evidence is available on the association between meteorological factors and the occurrence of influenza among children in subtropical regions. The current study aimed to explore the effects of meteorological factors on influenza among children in Guangzhou, a subtropical city in China.

**Methods:**

The distributed lag nonlinear model (DLNM) was used to assess the effects of meteorological factors on children influenza occurrence in Guangzhou, China. Daily number of influenza cases among children aged 0‐17 years from 2013 to 2017 were obtained from the National Information System for Disease Control and Prevention.

**Results:**

Mean temperature, relative humidity, and atmospheric pressure were associated with influenza cases. The relative risks (RRs) increased as temperature fell below 20°C. The relationship between relative humidity and influenza cases could be described with a U‐shaped curve, and the RRs increased if relative humidity was lower than 50% or higher than 80%. The risk of influenza increased with rising atmospheric pressure with 1005 hPa as the break point. The cold effect, humid effect, dry effect, high‐pressure effect, and low‐pressure effect showed statistical significance both in female and male. The cold effect increased with age. The humid‐effect affects all age ranges of children, but dry effect mainly affected 4‐14 years old. High‐pressure effect mainly affected the 0‐3 years old, whereas low‐pressure effect protected preschool children aged 0‐6 years old.

**Conclusion:**

Mean temperature, relative humidity, and atmospheric pressure might be important predictors of the influenza occurrence among children in Guangzhou.

## 1 INTRODUCTION

Influenza is a global public health concern; annual epidemics attack 3‐5 million people of all ages and contributes to 500 000 deaths worldwide.^1^ Children aged 6‐59 months face the highest risk of complications. In temperate regions, seasonal epidemics occur mainly in winter. While in tropical and subtropical areas, irregular epidemics may break out throughout the year.^1^, ^2^ In southern China, influenza is prevalent throughout the year; it has a clear peak in summer and a less pronounced peak in winter.^3^ Hong Kong, close to Guangzhou city, experiences semi‐annual cycles with epidemics usually peaking in spring and summer.^4^, ^5^ The seasonality of influenza suggests that meteorological factors might influence the spread of the disease.

Several studies have suggested an association between climate change and influenza spread, but the findings are inconsistent.^6^, ^7^, ^8^ Experimental and modeling studies have suggested that cold temperature and low relative/absolute humidity increase the risk of influenza transmission and survival in temperate settings,^3^, ^4^, ^5^, ^9^ while rainfall fluctuations may drive influenza activity in low latitudes, but this has not been well documented.^5^, ^10^ In tropical and subtropical regions, where the annual average temperature and humidity are much higher, there is insufficient evidence to prove a relationship between influenza risk and temperature or humidity. Furthermore, since temperature, precipitation, humidity, and other parameters may vary significantly within a climatic zone, it needs to explore the local information on influenza seasonality.

In Guangzhou, a subtropical city in China which often experiences extreme weather, the effects of temperature with low humidity and other meteorological factors on influenza transmission in children are not clearly understood. In this study, we established a distributed lag nonlinear model (DLNM) with the daily time series data during 2013‐2017 to assess the effects of meteorological factors on the occurrence of influenza among children in Guangzhou. We also examined whether the association was regulated by age and sex. The findings of this study may provide evidence for health authorities to develop an early warning system for influenza epidemic, so as to limit the health impacts on children.

## 2 METHODS

### 2.1 Setting

**Figure 1:**
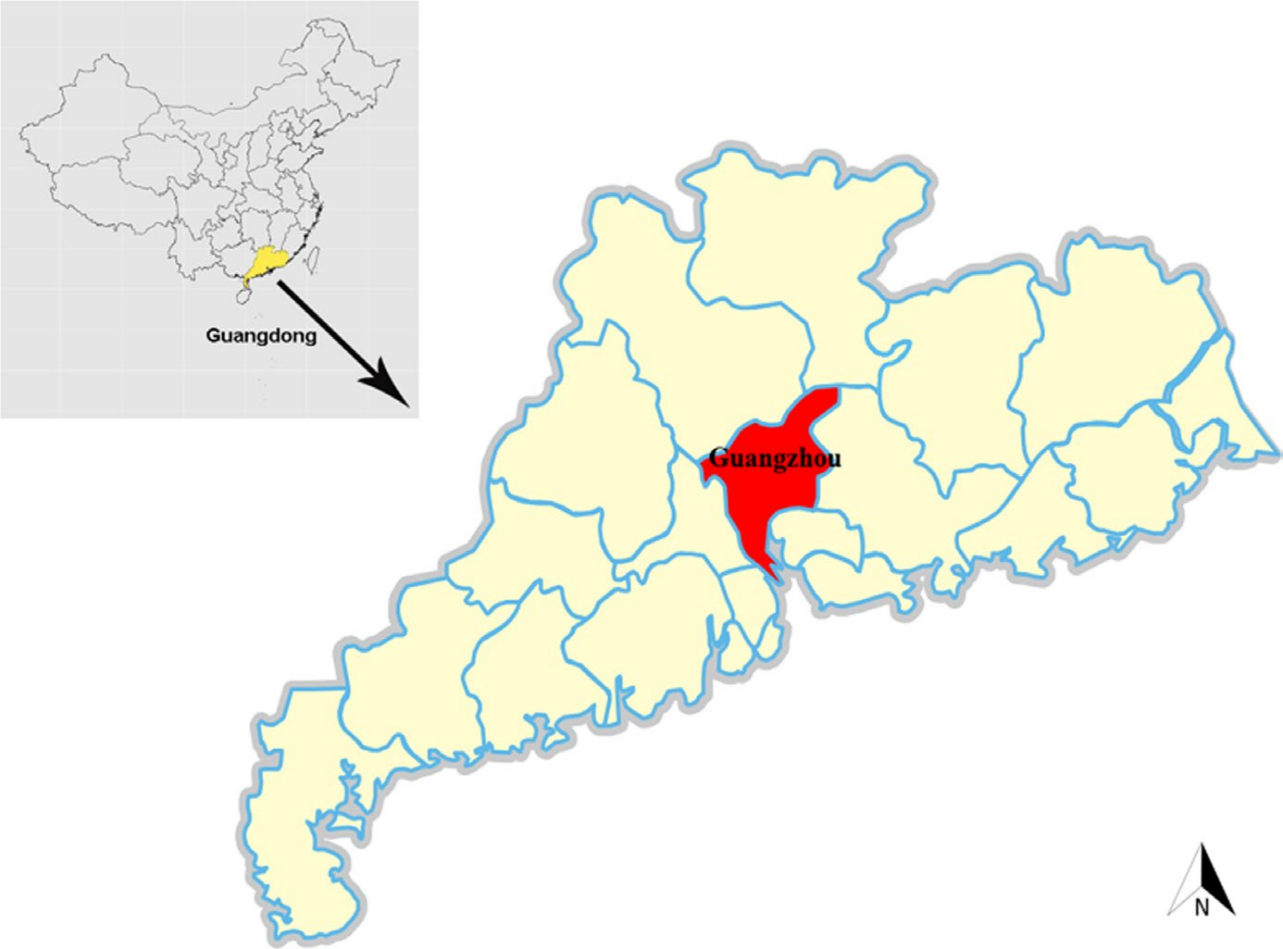
Geographical location of the study area in China

Guangzhou, the capital of Guangdong Province in southern China, is located at 22°26′N to 23°56′N and 112°57′E to 114°3′E.^11^, ^12^ Covering an area of 7434 square kilometers and 11 districts, Guangzhou has a population of 14.0 million in 2017, including 1.71 million (12.21%) aged 0‐14 years, 12.33 million (87.79%) aged over 15 years. The monsoon in subtropical Asia gives Guangzhou a long, hot, and humid summer, and a short, mild, and dry winter. January and February have the lowest mean temperatures (6.5‐12.1°C). Extremely wet days, locally known as “Hui‐nan‐tian” in February and March, appear when the dew point temperature of the outdoor air exceeds the surface temperature of indoor objects, along with high air humidity up to 100%.^13^, ^14^ The period between May and September is hot and humid, with the mean temperature (33.0‐34.9°C) peaking between July and August. The average annual rainfall ranges from 1370 to 2353 mm and exhibits a seasonality.^15^ Figure 1 shows the geographical location of Guangzhou.

### 2.2 Data collection

Influenza is a notifiable disease in China and categorized as a Class “C” infectious disease. According to China's notifiable infection disease regulations, all influenza cases should be reported to the National Information System for Disease Control and Prevention maintained by the Chinese Center for Disease Control and Prevention (China CDC, Beijing).^16^ Daily data on the cases of influenza among children aged 0‐17 years from January 1, 2013 to December 31, 2017, in Guangzhou were obtained from the national surveillance network. The data from this network were collected from 214 sentinel hospitals and community medical service centers.

According to the clinical criteria in a guidebook published by the Chinese Ministry of Health in 2011,^17^ influenza cases were diagnosed based on clinical symptoms with fever of ≥38°C and the acute onset of respiratory symptoms without an alternate explanation when influenza viruses were circulating in the community, and/or respiratory samples tested positive for influenza. The influenza cases were strictly examined and verified by various levels of CDC. At least 5‐10 nasopharyngeal/throat swab specimens, randomly from the patients within first 3 days after onset and who hadn't received antiviral treatment, were prepared weekly in each sentinel setting. Serotyping and sequencing were performed at provincial surveillance laboratories with quality control from Guangdong Province Center for Disease Control and Prevention (GDCDC).

Demographic information of all cases included gender, age (between the date of influenza onset and the date of birth), and the form of child care. According to our data, more than 83.08% influenza cases were children aged 0‐17 years. Therefore, we focused on the cases among children aged 0‐17 years in this study. The form of child care might impact influenza infection in children. According to our data, children aged 0‐3 years are usually cared for at home, 4‐6 years attend kindergarten, and 7‐14 years go to primary and middle schools, 15‐17 years go to senior school. To investigate which subpopulation was most susceptible to meteorological changes, we conducted the analyses for different age subgroups (0‐3, 4‐6, 7‐14, and 15‐17 years) and sex (male and female).

The daily meteorological data including maximum, mean, and minimum temperature, and relative humidity, daily precipitation, wind velocity, and atmospheric pressure were obtained from Guangdong Meteorological Administration.

### 2.3 Data analysis

We used Pearson correlation analysis to examine the correlation between daily meteorological factors and influenza cases. Scatter plots were drawn to observe the patterns of meteorological factors and influenza cases over time. Since the daily number of influenza cases follows a Poisson distribution, and the meteorological factors such as temperature have lag effects, we used a distributed lag nonlinear model (DLNM) to assess the association between meteorological factors and the daily number of influenza cases.^12^, ^18^ DLNM is a flexible model that simultaneously estimates the nonlinear and delayed exposure‐response relationship, especially the effects of meteorological factors on health.^18^

We calculated Pearson's correlation coefficients matrix within the meteorological factors for a preliminary analysis. Because of the strong correlation coefficient between temperature and atmospheric pressure (Table 2), we developed two DLNMs to independently analyze the two variables. A Poisson regression with quasi‐Poisson function was established to test the over‐dispersion in the daily number of influenza cases. All models were adjusted with indicator variables, like time trend, day of week (DOW), and public holidays. The calculation was as follows:

$$\begin{array}{cc} \text{Log}[E\left(Y_{t}\right)]= & \alpha +NS\left(M,df,lag,df\right)+\sum NS_{i}\left(X_{i},df,lag,df\right)+\gamma {\text{DOW}}_{i}+\\ & \delta {\text{Holiday}}_{t}+NS\left(\text{Time},6/\text{year}\right) \end{array}$$

In this equation, *Y*_t_ is the daily number of influenza cases on day t; α is intercept; NS is a natural cubic spline; *M* is one of the two strongly correlative variables to estimate including temperature (or atmospheric pressure); *df* is the degree of freedom; *X*_i_ is the other explanatory variables of meteorological factors including relative humidity and atmospheric pressure; DOW_t_ is day t of the week, and γ is vector of coefficients; Holiday_t_ is a binary variable the value of which is “1” if day t is a public holiday; Time refers to duration of seasonality in the calendar year and long‐term trend, and we found that using a NS with 6 *df* per year for time produced the best model fitting based on the Akaike Information Criterion for quasi‐Poisson (Q‐AIC), which was supported by other references.^11^, ^18^

We used the Q‐AIC to choose the *df* for the meteorological factors and the maximum lag. The effects of daily mean temperature, relative humidity, and atmospheric pressure were controlled using NS of 2 *df*, 2 *df* and 3 *df*, and the maximum lag of all 27 days, respectively. The reference level was defined as the median value of mean temperature, relative humidity, and atmospheric pressure to calculate the relative risks (RRs) and 95% confidence intervals (CI).

The extreme effects of high value at the 95^th^ percentile and extreme low value at the 5^th^ percentile were estimated. The relative risks (RRs) of influenza brought by high temperature (hot effect), high relative humidity (humid effect), and high pressure (high‐pressure effect) were calculated comparing the 95^th^ percentile to the median value. The relative risks (RRs) of influenza brought by low‐temperature (cold effect), low relative humidity (dry effect), and low‐pressure (low‐pressure effect) were calculated by comparing the 5^th^ percentiles to the median value. To estimate the cumulative extreme effects of mean temperature, on the basis of Akaike Information Criterion (AIC) and other references,^11^, ^18^ a maximum lag of 10 days was selected for hot effect, of 27 days for cold effect, for humidity, a maximum lag of 27 days was selected for humid effect, of 20 days for dry effect, and for atmospheric pressure, a maximum lag of 15 days was selected for high‐pressure effect, of 20 days for low‐pressure effect.

All statistical tests were two‐sided, and *P*‐value < 0.05 was considered statistically significant. All analyses were performed using the R software (version 3.0.2; R Development Core Team 2013), and DLNM was carried out using the “dlnm” package.

## 3 RESULTS

A total of 58 840 children aged 0‐17 years were reported to have influenza in Guangzhou during January 1, 2013 and December 31, 2017, with a male to female sex ratio of 1.37:1(34 001:24 839). Among them, 25 395 cases (43.16%) were aged 0‐3 years old; 15 285 cases (25.98%) aged 4‐6 years old; 16 818 cases (28.58%) aged 7‐14 years old, and 1342 cases (2.28%) aged 15‐17 years old.

Daily meteorological conditions and influenza cases are shown in Table 1. The median number of influenza cases was 10, and the daily average maximum, mean, and minimum temperatures were 26.71, 22.34, and 18.84°C, respectively. The average of relative humidity, daily precipitation, wind velocity, atmospheric pressure was 78.04%, 6.46 mm, 4.27 hours, 1004.97 hPa, respectively.

**Table 1:** Daily meteorological conditions and influenza cases of children in Guangzhou, 2013‐2017

Variables	Mean	SD	Min.	P25	P50	P75	Max.
Daily influenza frequency	32.22	60.52	0	3	10	30	497
Maximum temperature(°C)	26.71	6.29	5.50	22.20	27.80	32.00	38.30
Mean temperature (°C)	22.34	6.17	3.60	17.50	23.80	27.50	33.50
Minimum temperature (°C)	18.84	6.27	1.20	13.78	20.60	24.30	28.40
Relative humidity (%)	78.04	12.09	26.00	72.00	80.00	86.00	100.00
Daily precipitation (mm)	6.46	16.39	0	0	0	4.10	164.10
Wind velocity (m/s)	2.28	0.99	0.60	1.60	2.00	2.80	7.50
Atmospheric pressure (hPa)	1004.97	6.73	985.3	999.80	1004.60	1010.30	1027.40
Sun (h)	4.27	3.80	0	0	3.80	8.10	12.00

**Figure 2:**
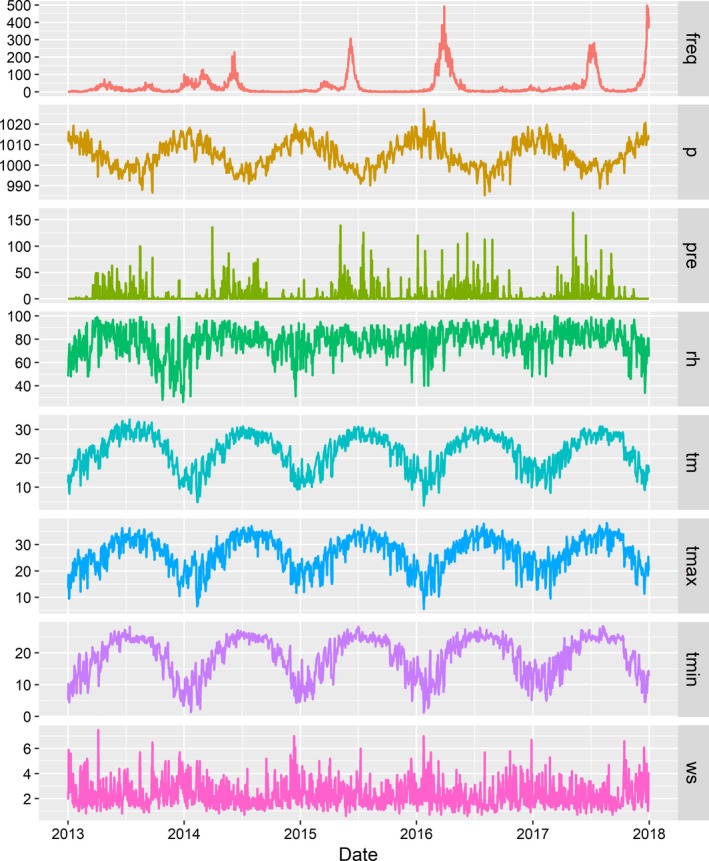
The time series of daily meteorological factors and daily influenza cases in Guangzhou, 2013‐2017 (freq: Daily influenza cases, p: Atmospheric pressure (hPa), pre: daily precipitation(mm), rh: Relative humidity (%), tm: Mean temperature (°C), t max: Maximum temperature (°C), t min: Minimum temperature (°C), Ws: Wind velocity (m/s))

Figure 2 shows the time series of daily meteorological factors and daily influenza cases in Guangzhou, 2013‐2017. There was an obvious seasonal pattern and upward trend in the number of influenza cases for children aged 0‐17 years. The time points of seasonal peaks were not stable, arising in spring (April‐May) in 2013, in winter lasting to summer (January‐June) in 2014, in late spring and early summer (May‐June) in 2015, in spring (March‐April) in 2016, in summer (June‐July) and winter (December) in 2017.

Table 2 provides the matrix of Pearson's correlation coefficients within the meteorological factors. For all age groups, the daily influenza cases were positively correlated with sunshine hours only. However, in the further stratified analyses by age and sex, temperature, humidity, and air pressure were significantly correlated with influenza cases. Thus, we used the statistically significant variables above (mean temperature, relative humidity, and atmospheric pressure) for the subsequent analyses. Meanwhile, there was a high correlation between temperature and atmospheric pressure (correlation coefficient 0.86, *P *<* *0.05). Thus, temperature and atmospheric pressure were not included in the same model due to the potential collinearity.

**Table 2:** Pearson correlation coefficients matrix of meteorological factors on influenza cases of children in Guangzhou, 2013‐2017

Variables	Influenza frequency	Maximum temperature (°C)	Mean temperature (°C)	Minimum temperature (°C)	Relative humidity (%)	Precipitation (mm)	Wind velocity (m/s)	Atmospheric pressure (hPa)
Influenza frequency	1							
Maximum temperature(°C)	−0.02	1						
Mean temperature(°C)	−0.02	0.95*	1					
Minimum temperature (°C)	0.00	0.87*	0.96*	1				
Relative humidity (%)	0.08	0.16*	0.26*	0.42*	1			
Daily precipitation (mm)	0.04	0.06	0.12*	0.19*	0.39*	1		
Wind velocity(m/s)	−0.04	−0.36*	−0.31*	−0.28*	−0.30*	−0.03	1	
Atmospheric pressure(hPa)	−0.02	−0.79*	−0.86*	−0.87*	−0.42*	−0.27*	0.23*	1
Sun (h)	−0.14*	0.42*	0.24*	0.05	−0.53*	−0.28*	−0.02	−0.06

* *P *<* *0.05.

**Figure 3:**
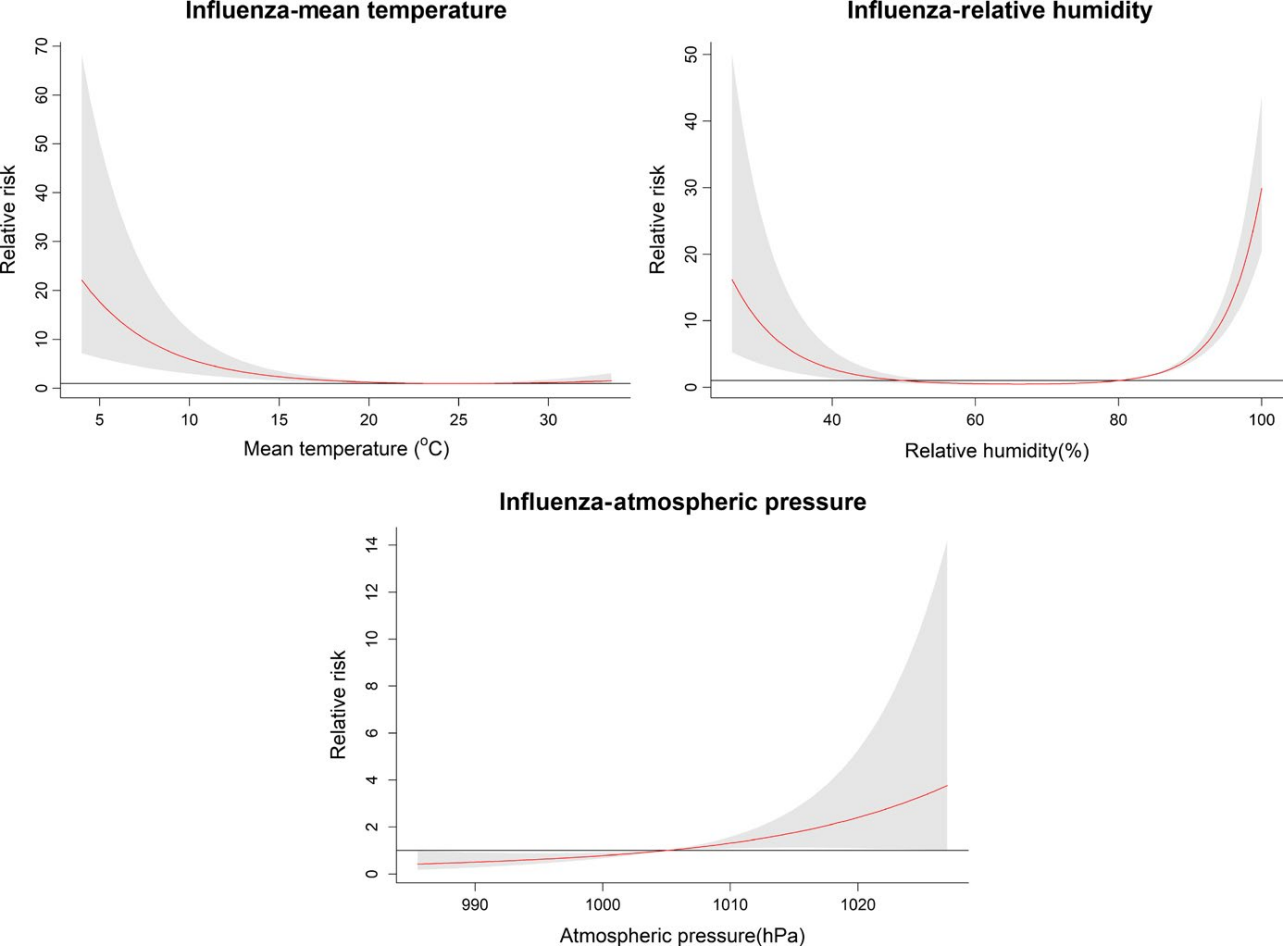
The estimated overall effects of mean temperature, relative humidity, and atmospheric pressure on children influenza cases over the corresponding lag days. Confounding factors included time trend, DOW, and public holidays. The red lines represent mean relative risks, and gray regions represent 95% confidence intervals. The median value of each meteorological factor (mean temperature: 23.8°C, humidity: 80%, pressure:1005 hPa) is as a reference level

For a better interpretation, the relative risks (RRs) and 95% confidence intervals (CI) of daily influenza occurrence (relative to the risk at the median value of the covariate) were plotted against covariate values for mean temperature, relative humidity, and atmospheric pressure over the corresponding lag days in Figure 3. In general, mean temperature, relative humidity, and atmospheric pressure were associated with influenza cases. The relative risks (RRs) increased as temperature fell below 20°C. The relationship between relative humidity and influenza cases could be described with a U‐shaped curve. The RRs increased if relative humidity was lower than 50% or higher than 80%. The risk of influenza increased with rising atmospheric pressure with 1005 hPa as the break point.

**Figure 4:**
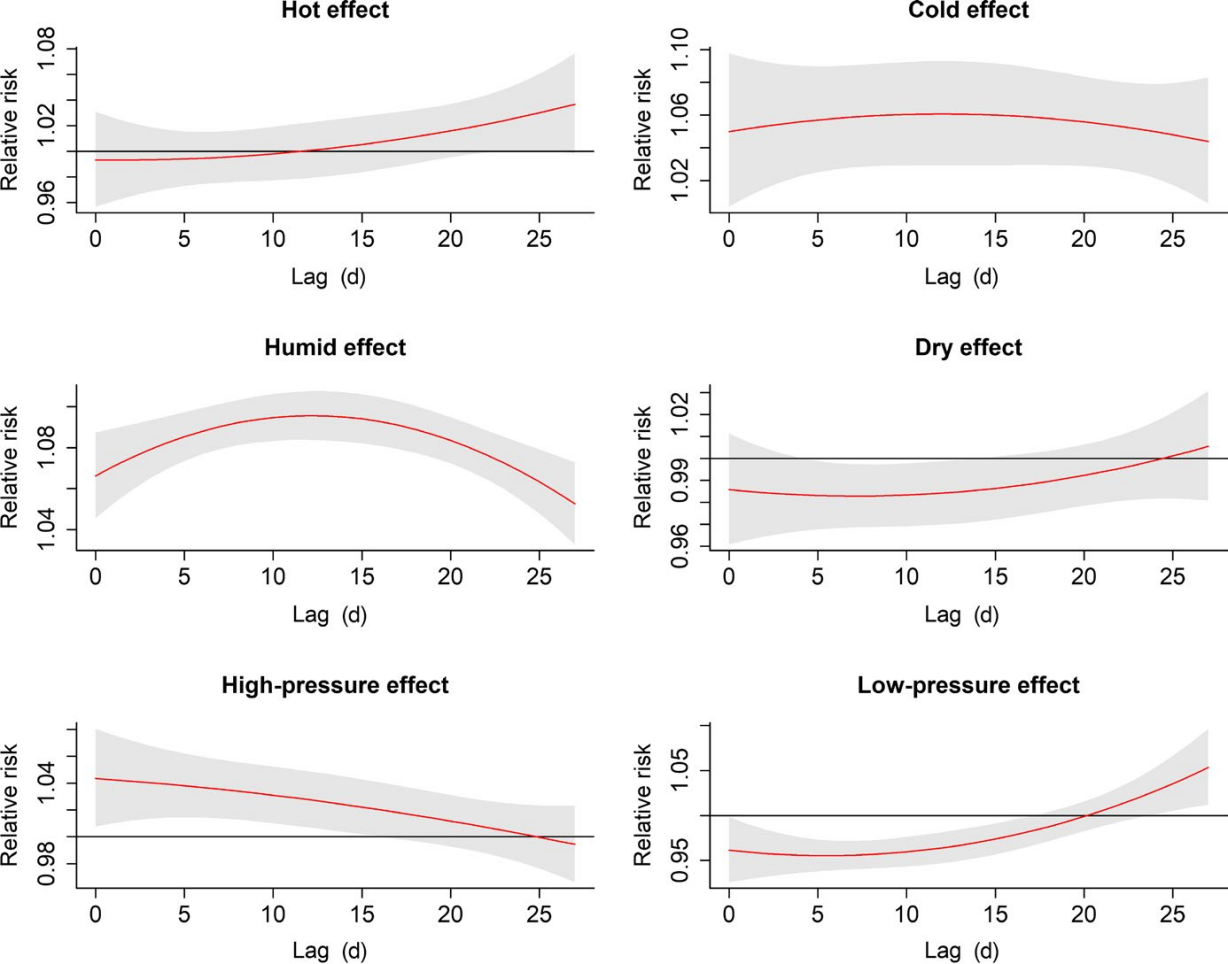
The estimated extreme effects at the 95th and the 5th percentile of mean temperature, relative humidity, and atmospheric pressure at different lag days on children influenza cases. The median value of each meteorological factor (mean temperature: 23.8°C, humidity: 80%, pressure: 1005 hPa) is as a reference level. The red lines are mean relative risks, and gray regions are 95% confidence intervals

To identify the extreme effects, the estimated effects of mean temperature, relative humidity, and atmospheric pressure comparing the 95^th^ percentile to the median value and the 5^th^ percentiles to the median value were plotted in Figure 4. The hot effect was not significant, whereas cold effect was a significant risk factor occurring within 0‐27 lag days. A significant humid effect was observed along 0‐27 lag days, but the dry effect was not significant. The high‐pressure effect peaked at the current day and appeared within 0‐15 lag days. The low‐pressure effect appeared within 0‐20 lag days.

We also calculated the cumulative extreme effect of meteorological factors on influenza (Table 3). For all age children, the RR of hot effect at 10^th^ lag day, humid effect at 27^th^ lag day, and high‐pressure effect at 15^th^ lag day were 0.99 (95% CI: 0.92, 1.06), 1.45 (95% CI: 1.39, 1.51), 1.11 (95% CI: 1.02, 1.20) comparing the 95th to the 90th percentile of their own, respectively. The RR of cold effect at 27^th^ lag day, dry effect at 20^th^ lag day, and low‐pressure effect at 20^th^ lag day were 1.63 (95% CI: 1.41, 1.88), 1.28 (95% CI: 1.11, 1.47), 0.58 (95% CI: 0.50, 0.66) comparing the 5th to the 10th percentile of their own, respectively.

**Table 3:** The cumulative effects of meteorological factors on influenza cases of children by sex and age

Variables	Mean temperature (°C)		Relative humidity (%)		Atmospheric pressure (hPa)	
	Cumulative extreme hot effect (95% CI)	Cumulative extreme cold effect (95% CI)	Cumulative extreme humid effect (95% CI)	Cumulative extreme dry effect (95% CI)	Cumulative extreme High‐Pressure effect (95% CI)	Cumulative extreme Low‐Pressure effect (95% CI)
All age children	0.99 (0.92, 1.06)	1.63 (1.41, 1.88)	1.45 (1.39, 1.51)	1.28 (1.11, 1.47)	1.11 (1.02, 1.20)	0.58 (0.50, 0.66)
Sex						
Male	0.97 (0.90, 1.05)	1.62 (1.39, 1.89)	1.44 (1.38, 1.50)	1.32 (1.13, 1.53)	1.10 (1.00, 1.20)	0.57 (0.49, 0.66)
Female	1.01 (0.93, 1.10)	1.64 (1.39, 1.94)	1.47 (1.40, 1.54)	1.22 (1.04, 1.43)	1.12 (1.02, 1.23)	0.59 (0.50, 0.69)
Age						
0‐3 y	0.93 (0.86, 1.01)	1.51 (1.27, 1.79)	1.43 (1.36, 1.50)	1.15 (0.95, 1.39)	1.16 (1.04, 1.29)	0.52 (0.45, 0.60)
4‐6 y	0.92 (0.84, 1.00)	1.45 (1.21, 1.73)	1.39 (1.32, 1.47)	1.39 (1.18, 1.64)	1.09 (0.99, 1.20)	0.60 (0.50, 0.72)
7‐14 y	1.09 (0.95, 1.26)	1.82 (1.43, 2.32)	1.55 (1.44, 1.68)	1.38 (1.09, 1.75)	1.06 (0.92, 1.23)	0.77 (0.55, 1.07)
15‐17 y	1.08 (0.83, 1.41)	2.92 (1.59, 5.35)	1.29 (1.09, 1.54)	0.73 (0.35, 1.54)	1.08 (0.74, 1.59)	0.74 (0.39, 1.39)

The effects of an extreme high value (hot effect, humid effect and high‐pressure effect) are presented by the relative risk (RR) and 95% confidence interval (CI) of the meteorological factors on children influenza cases by comparing the 95th to the 90th percentile of their own effect (for all age children, the reference level of hot effect was 29.2°C, humid effect 92%, high‐pressure effect 1014.10 hPa). The maximum lag day of 10 d was selected for hot effect, of 27 d for humid effect, of 15 d for high‐pressure effect.

The effects of an extreme low value (cold effect, dry effect and low‐pressure effect) are presented by the relative risk (RR) and 95% confidence interval (CI) of the meteorological factors on children influenza cases by comparing the 5th to the 10th percentile of their own (for all age children, the reference level of cold effect was 12.96°C, dry effect 63%, low‐pressure effect 996.60 hPa). The maximum lag day of 27 d was selected for cold effect, of 20 d for dry effect and of 20 d for low‐pressure effect.

For the subpopulations, the cold effect, humid effect, dry effect, high‐pressure effect, and low‐pressure effect showed statistical significance both in female and male populations. The RR of cold effect increased with age, whereas the humid effect did not present with a similar trend. The significant dry effect was mainly among 4‐6 years group (RR = 1.39, 95% CI: 1.18, 1.64) and 7‐14 years group (RR = 1.38, 95% CI: 1.09, 1.75). The significant high‐pressure effect was among 0‐3 years group (RR = 1.16, 95% CI: 1.04, 1.29), and the significant low‐pressure effect, which was protective, was among 0‐3 years group (RR = 0.52, 95% CI: 0.45, 0.60) and 4‐6 years group (RR = 0.60, 95% CI: 0.50, 0.72).

## 4 DISCUSSION

To the best of our knowledge, this is the first in China to apply a DLNM model and use population‐wide monitoring data to evaluate the effects of meteorological factors on influenza occurrence among children in a subtropical city. In addition to temperature and humidity which were often mentioned in previous studies,^6^, ^7^, ^19^ the role of other potential meteorological factors including daily precipitation, wind velocity, and atmospheric pressure were also explored in the present study. Our study showed that the correlation between relative humidity and influenza cases was illustrated with a U‐shaped curve, and influenza cases increased with colder temperature and higher pressure.

Our study indicated that influenza cases increased significantly with a low temperature below 20°C, which is consistent with many previous studies.^5^, ^6^, ^10^, ^19^, ^20^, ^21^ For example, a study using a guinea pig model found the most efficient transmission of influenza virus was at 5°C and the most inefficient transmission at 30°C.^22^ Two studies from tropical and subtropical areas showed that cool climate (14‐19°C in Hong Kong, China,^23^ and 13‐22°C in Niamey, Niger^20^) raised the influenza occurrence. One of the possible reasons for the increased influenza cases in Guangzhou is that the cool winter temperature lowers the activity of influenza virus and promotes the transmission of aerosol virus indoors. The findings can strengthen the awareness that indoor air must be ventilated with outdoor air to reduce the transmission of influenza virus.

Previous studies found dry weather was associated with the seasonal influenza epidemic in winter in temperate areas,^4^, ^5^, ^6^, ^9^ but this association was not significant in tropical and subtropical areas such as Hong Kong.^5^, ^7^, ^10^, ^20^, ^23^ Our investigation verified a U‐shaped relationship between the relative humidity and influenza cases, which is similar to the results of some previous experimental and epidemiological studies.^5^, ^24^, ^25^ The influenza virus is active when the relative humidity is below 50%, especially between 20% and 35%.^6^ Influenza virus can attack the innate defense of host nasal epithelia, and it is more productive and transmittable (via respiratory aerosols and droplets) in dry weather conditions.^6^, ^9^, ^25^ Our findings indicate that it will be useful to humidify the indoor air to curb the spread of influenza virus in dry winter.

Influenza cases normally peak in winter in temperate regions,^3^, ^5^ but the influenza peak appears between January and June in Guangzhou, Hong Kong and some other tropical and subtropical cities.^4^, ^5^, ^23^ The spring and summer of Guangzhou are warm, humid and rainy, with occasional extreme humid wet days. High relative humidity or precipitation might swing the seasonality of influenza epidemics.^5^, ^7^, ^10^, ^23^, ^26^ The effect of humidity on influenza cases was verified by our investigation. High humidity can bring forth droplets that bind to influenza virus, increasing the concentration of virus in the air around the infection source. Experimental study has shown stronger infectivity of influenza virus in a high relative humidity (60%‐80%)^22^. Under such conditions, air‐borne transmission becomes more efficient as air humidity decreases. Children, especially school children, spend most of their time indoors. Dehumidifying the air in spring and summer, especially on wet days in Guangzhou may reduce the influenza virus. Moreover, our study showed that the effect of extreme wetness (100% relative humidity) in spring was obvious.

Little literature is available about the effects of atmospheric pressure on influenza. We found that the number of influenza cases increased under high pressure (upper 1005 Pa), most obviously among 0‐3‐year‐old children. Similarly, Soebiyanto et al reported that the incidence of influenza was correlated with maximum atmospheric pressure in Maricopa County.^4^ High pressure often means a sunny day when children's outdoor activities increase the risk of virus infection.^27^ However, our investigation found that low atmospheric pressure protected preschool children aged 0‐6 years old. Guangdong is a coastal province frequented by tropical cyclones, mostly typhoons, which bring low pressure and rich rainfall.^18^, ^28^, ^29^ Low pressure can also reduce children's outdoor activities and interpersonal body contacts.

Several limitations exist in this study. First, the meteorological data were for outdoor measurements, but children spend most of their daytime in air‐conditioned rooms where the humidity might be different from that of the outside. Second, other potentially influential factors (such as school closure, vaccination, economy) were not analyzed in our investigation.^8^, ^30^ Third, this is a single city study and more cities in different areas should also be covered.

## 5 CONCLUSIONS

Mean temperature, relative humidity, and atmospheric pressure might be important predictors of the influenza occurrence among children in Guangzhou. Accordingly, the warning system for influenza should be improved to protect this population.

## COMPETING INTERESTS

The authors declare that they have no competing interests.

## AUTHOR CONTRIBUTORS

Qiaozhi Guo and Zhiqiang Dong are joint first authors. Danyang Zhao obtained funding. Zhiqiang Dong, Tiegang Li, Weilin Zeng, Wensui Hu, and Jianpeng Xiao collected the data and were involved in data cleaning, and verification. Weilin Zeng and Qiaozhi Guo analyzed the data. Qiaozhi Guo, Wenjun Ma, Danyang Zhao, Xin Sun, Sitang Gong contributed to the interpretation of the results and critical revision of the manuscript for important intellectual content and approved the final version of the manuscript. All authors have read and approved the final manuscript.

## ETHICS APPROVAL

This study is approved by the ethical review committee of the Guangzhou Center for Disease Control and Prevention, Guangdong Provincial Institute of Public Health, and Guangzhou Women and Children's Medical Center. Data collected in this study are maintained with utmost confidentiality and anonymization for reporting.

## DATA SHARING

No additional data is available.
